# Gender differences in response to abdominal compartment syndrome in rats

**DOI:** 10.1186/s13104-019-4353-6

**Published:** 2019-06-08

**Authors:** Or Barkai, Ahmad Assalia, Evgeny Gleizarov, Ahmad Mahajna

**Affiliations:** 1 Department of General Surgery, Laboratory of Shock and Trauma Research, Rambam Medical Center and, The Bruce Rappaport Faculty of Medicine, Technion–Institute of Technology, P.O. Box 9602, 31096 Haifa, Israel; 2 The Department of Urology, Rambam Medical Center and, The Bruce Rappaport Faculty of Medicine, Technion–Institute of Technology, Haifa, Israel

**Keywords:** Abdominal compartment syndrome (ACS), Intra-abdominal pressure (IAP), Female/male, Trauma

## Abstract

**Objective:**

Our study aims to emphasize the novelty of female rats in regard to their hemodynamic changes in response to abdominal compartment syndrome. A group of 64 rats was randomly divided into 4 subgroups for each gender. Except for the control, intra-abdominal pressure was increased to 10, 20, 30 mmHg. Survival time, mean arterial pressure, pH and lactate were determined at different time intervals.

**Results:**

As IAP was 20 mmHg, a statistically difference was seen between the male group and the female group starting from 15 min (126 ± 9.7 mmHg, 124 ± 14.7 mmHg respectively, p < 0.02) and lasting 2 h. At 30 mmHg, a statistically difference was seen between 30 to 60 min (p < 0.05). Only group 2 presented results with statistical power both at 30 and at 60 min concerning pH (p = 0.003, p < 0.001 respectively). In the lactate measurements at IAP of 10 mmHg, at 60 min male lactate level was 3.93 ± 1.13 and 2.25 ± 0.33 in female rats (p = 0.034). Female rats that were subjected to IAP of 20 mmHg and 30 mmHg had significantly better survival than male rats that were subjected to the same pressure (p < 0.05 and p < 0.01, respectively). We concluded that female rats have better preserved their hemodynamic and metabolic parameters during ACS than male rats.

## Introduction

Acute and rapid elevation of Intra-abdominal pressure (IAP) exceeding 15 mmHg is considered to be pathologically elevated and has been termed intra-abdominal hypertension [1]. Abdominal compartment syndrome (ACS) is defined as an increased IAP ( > 20 mmHg) in combination with a single or multiple organ dysfunction which was not previously present. ACS is most commonly diagnosed in patients sustaining abdominal or pelvic trauma, or suffering some other intra-abdominal hemorrhagic catastrophe [2–4]. Sustained elevation of IAP causes increased intra-thoracic pressure and abnormalities in pulmonary dynamics, increased afterload, decreased venous return, decreased cardiac output, and decreased perfusion to the kidneys and intestinal mucosa [5–7].

Several clinical studies have investigated the relationship between gender and the development of sepsis and multiple organ failure after trauma. These studies have shown that incidence of morbidity and mortality from sepsis was higher in males [8–11]. Sex hormones are known to modulate both immunologic and physiologic functions in animal models in the setting of trauma-hemorrhage as well as humans under normal and stress conditions [12–17]. Although the precise mechanism of this sexual dimorphism remains unknown, studies have demonstrated that male sex hormones appear to be responsible for cellular and organ function depression [18, 19], while estradiol, the predominant sex hormone in females, has been shown to have protective effects [14, 20, 21].

We have developed a model of ACS in rats aiming to compare the hemodynamic and metabolic response to ACS in female and male rats and to assess the survival rate differences between the genders. We hypothesized that female rats will be better protected against lethal ACS than male animals.

## Main text

### Experimental animals

Experiments were performed in adult male and female Sprague–Dawley rats, weighing 250 to 350 g. Care and performance were carried out according to the National Research Council’s Guide for the Care and Use of Laboratory Animals, and approved by the Institutional Animal Care and Use Committee of the Technion Faculty of Medicine (reference number 002-01-2007).

### Experimental procedures

The animals were anesthetized by intramuscular injection of an anesthetic solution (0.3 mL/100 g body weight) containing 0.5 mg/mL dehydrobenzperidol (Janssen Pharmaceutica, Beerse, Belgium) and 8 mg/mL ketamine (Parke-Davis, Pontypool, Gwent, UK), and anesthesia was maintained by additional doses as necessary. The animals were kept supine during the experiments.

Polyethylene catheters (PE-50, Intramedic Medical Formulation, Clay-Adams, Parsippany, NJ) was introduced into the carotid artery for blood pressure and pulse measurements and blood sampling. The arterial line containing a calibrated pressure transducer (Cobe CDX III, Argon, Athens, TX) was directly connected to a Controlled Data Acquisition System (Cyber Amp 380, Axon Instruments, Foster City, CA). Heart rate was computed from the arterial tracing. After vascular cannulation, tracheostomy was performed and the rats were connected to mechanical ventilation. A rate of 60 cycles in minute and a tidal volume of 1.5 mL/100 g were maintained during the study. Mean arterial pressure (MAP) and peak inspiratory pressure were measured. Blood gases, pH, blood electrolytes and lactic acid were determined by an amperometric and some ion-selective electrode methods (Compact 2 AVL and 9180 Electrolyte Analyzer, AVL Medical Instruments, Shaffhausen, Switzerland).

### Abdominal compartment syndrome

IAP was induced by insertion of sterile latex powdered surgical glove (Triflex LP) into the peritoneal space by way of midline-laparotomy (3 cm incision). Both layers of the abdominal cavity were closed with a 3-0 silk continuous suture. The glove was connected by a Foley catheter—Latex (100% Silicone coated) to a fluid injection system and also to a pressure transducer (Cobe CDX III, Argon, Athens, TX). IAP was established at levels of 10 mmHg, 20 mmHg and 30 mmHg by instilling sterile normal saline through the catheter into the glove. The pressure remained constant throughout the experiment.

### Experimental protocol

Two groups, one consisting of 32 male rats and the other of 32 female rats, were randomly divided into 4 subsequent subgroups following anesthesia and cannulation. The designated groups were divided accordingly: Group 1 (n = 8) sham-operated including both cannulation and insertion of glove into the abdominal cavity, Group 2 (n = 8) IAP of 10 mmHg, Group 3 (n = 8) IAP of 20 mmHg, and Group 4 (n = 8) IAP of 30 mmHg. All groups were then observed for either 4 h or until death. The following parameters: MAP, pH, lactate level were analyzed prior to laparotomy. This took place at time 0, 15, 30, 60, 120, 180, and 240 min. The mean survival time in the 4 h observation period was computed. Following the 4 h observation period the surviving animals were euthanized by injection of an intravenous overdose of KCl 15%.

### Statistical analysis

Descriptive statistics in terms of mean, SD, median with 25–75% and ranges were performed to the whole parameters in the study. Normal distributions were tested by Shapiro–Wilk. AS a result of this test T-test or Mann Whitney U tests were used for differences between male vs female rats. p < 0.05 was consider as significant.

SPSS version 25 was used for the statistical analysis.

## Results

**Fig. 1:**
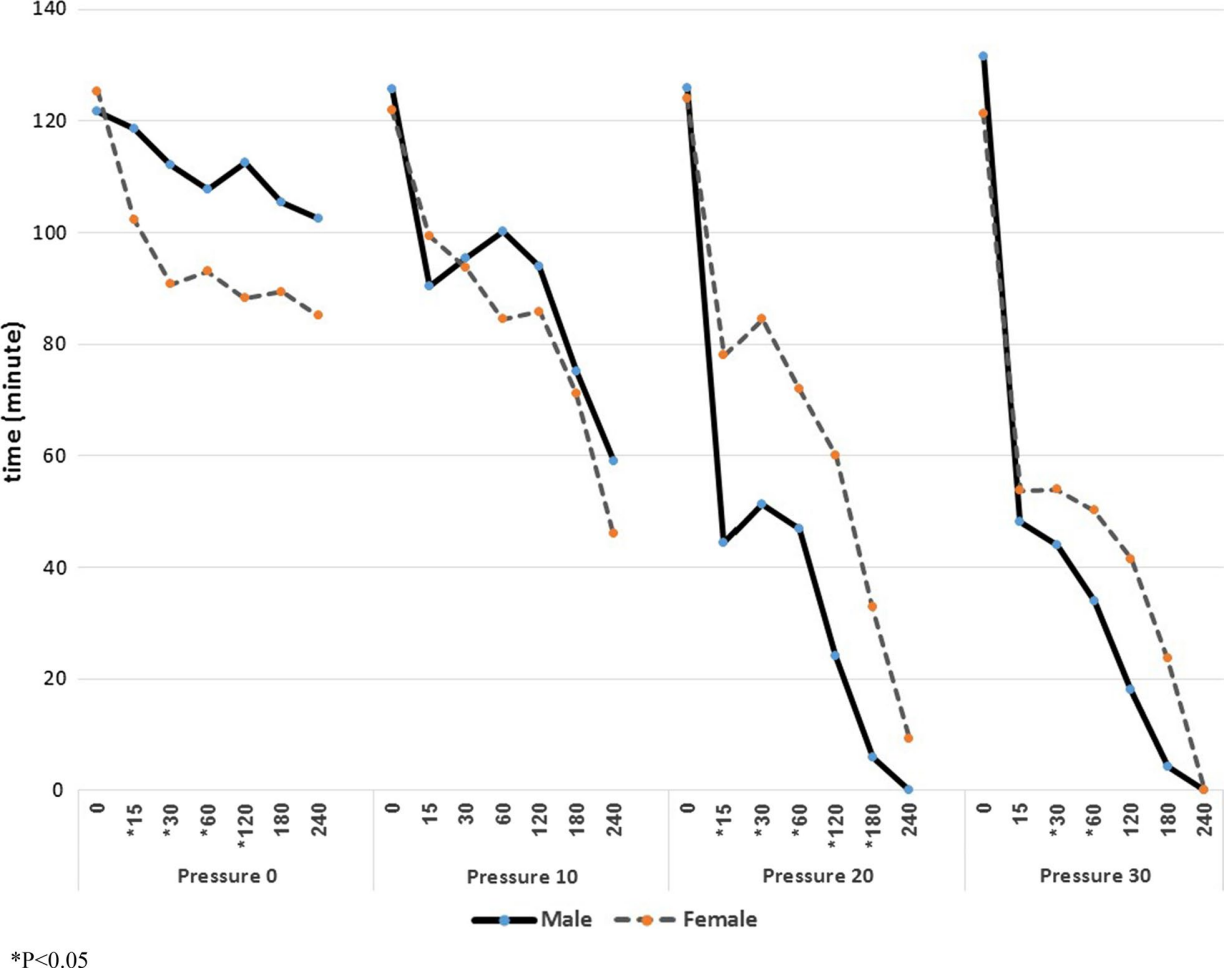
MAP of male and female at control group and exposed to abdominal compartment pressure

The control groups for both genders show normal values and deviation concerning all measurable categories. The female rats in the control group had a lower MAP (93 ± 5.9) to begin with compared to the control group of male rats (107 ± 15.9 mmHg). These results are statistically significant in the first 120 min (p < 0.03). When IAP was set at 10 mmHg, no statistical difference was seen between the male and the female groups. It is reasonable to assume that the decrease in the MAP in the male group was more pronounced than the female group, as the first had higher values to begin with. At an IAP of 20 mmHg, at time 0, there was no difference between the genders with MAP of 126 ± 0.9.7 in males and 124 ± 14.7 in females. After 15 min and a constant pressure maintained at 20 mmHg, a large difference was noted between the groups; MAP in the male group was 44 ± 9.1 while in the female group it was 78 ± 16.9 mmHg (p < 0.001). As time reached 120 min, there was still a statistically significant difference between the genders; male rats had an MAP of 24.22 ± 20.9 mmHg while the female rats had better hemodynamic results with MAP of 60 ± 15 mmHg. Establishing an IAP of 30 mmHg repeatedly showed no differences between the genders at time 0, however, progression to 30 and 60 min did show a statistically difference with values of 43.9 ± 9.9 and 33.9 ± 19.8 in male rats and 54 ± 9.3 and 50.2 ± 8.6 in female rats respectively (p < 0.044, p < 0.046) (Fig. 1).

**Fig. 2:**
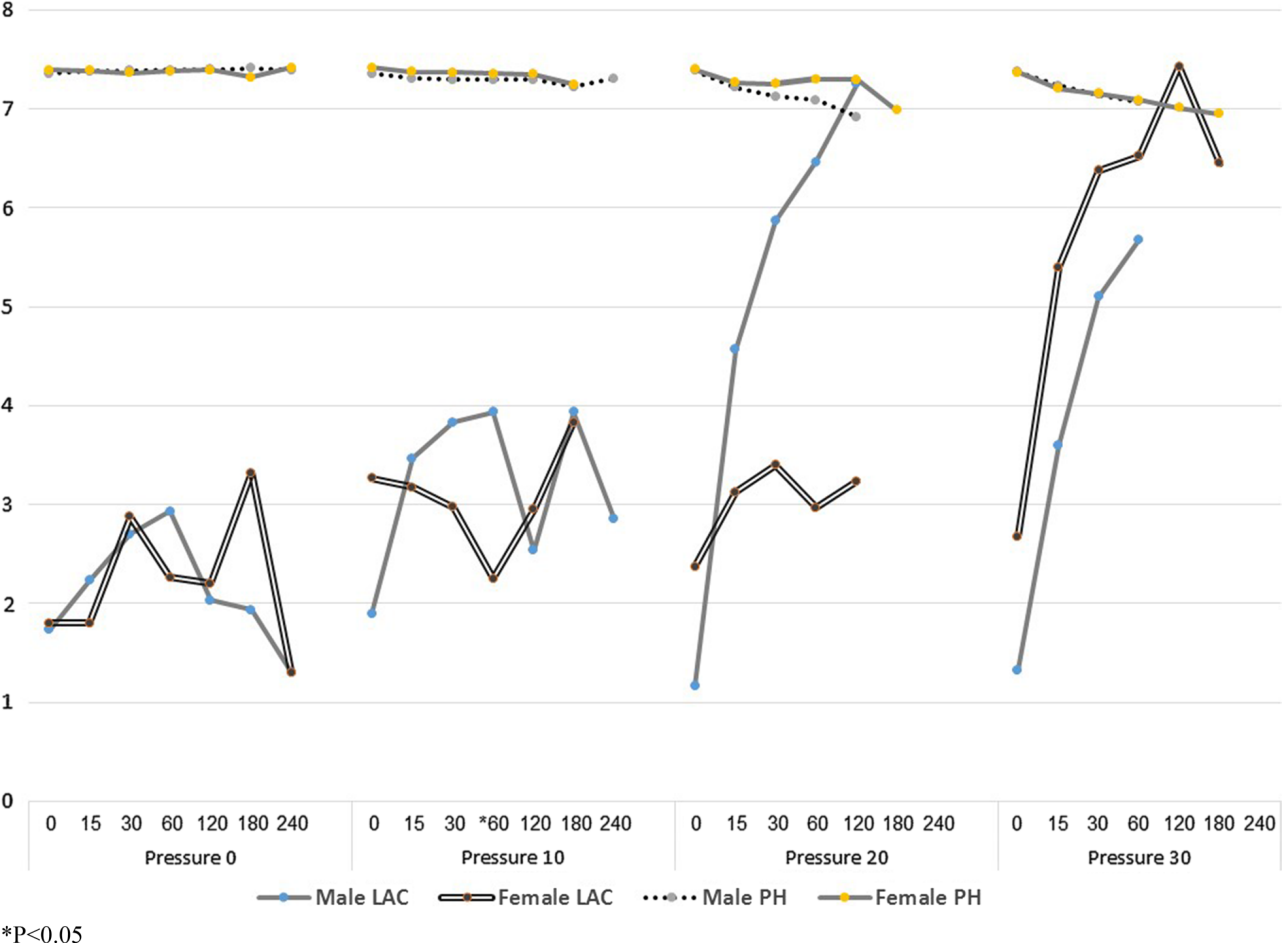
Blood lactate and pH level of male and female rats at control group and exposed to abdominal compartment pressure

Considering the pH results, only group 3 presented results with statistical power both at 30 and at 60 min. The pH of male rats at 30 min was 7.12 ± 0.75 and 7.08 ± 0.58 at 60 min while in the female rat group better results were measured; 7.25 ± 0.09 at 30 min and 7.29 ± 0.045 at 60 min (p = 0.003, p < 0.001 respectively) (Fig. 2).

When analyzing and comparing lactate measurements, the control group showed equivalent results between the genders, while subjects in group 2, at 60 min, with a maintained abdominal pressure at 10 mmHg, presented a statistically meaningful result with p value of 0.0034 as male lactate level was 3.93 ± 1.13 and 2.25 ± 0.33 in female rats (Fig. 2).

**Fig. 3:**
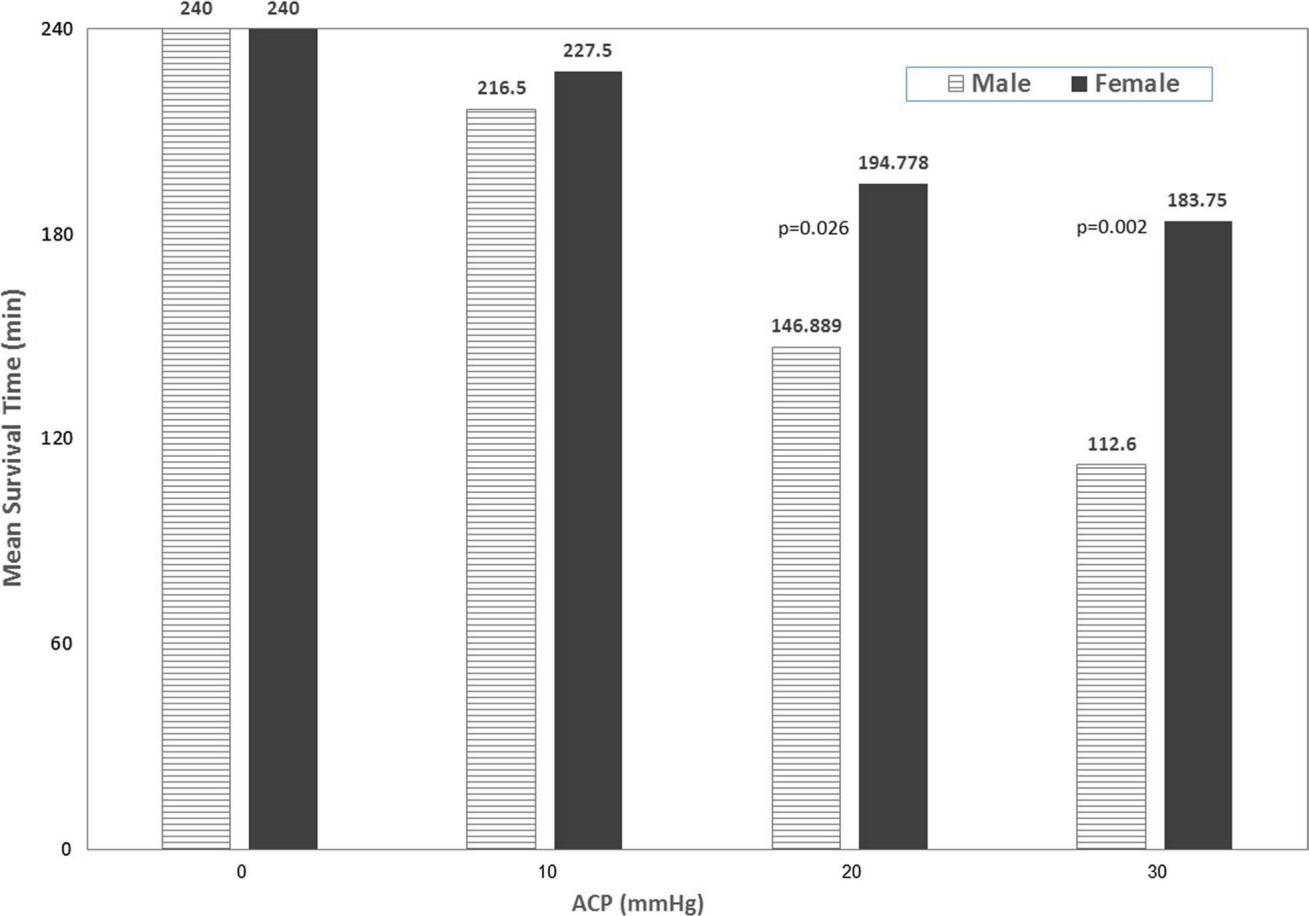
Survival rate of male and female rats exposed to abdominal compartment pressure

Survival rates at IAP of 10 mmHg were not statistically significant within the gender, however as the pressure increased, the dissimilarity was more pronounced. Female rats that were subjected to IAP of 20 mmHg and 30 mmHg had significantly better survival than male rats that were subjected to the same pressure (p < 0.05 and p < 0.01, respectively). In group 2, a single male rat demised before 180 min and 3 more at 240 min. In the same terms, 4 female subjects did not survive 240 min. At 20 mmHg, 6 males did not survive, 2 of them demised at 120 min, while the same number of females died, all passing the 120 min bar. At 30 mmHg, in group 4, none of the male rats survived beyond 180 min while 4 of the females did (Fig. 3).

## Discussion

ACS is defined as a sustained IAP that is associated with new organ dysfunction which generally occurs in patients who are critically ill or after emergency surgery [22, 23]. Failure to recognize the development of ACS causes tissue hypoperfusion, which may lead to multisystem organ failure, and potentially death. Mortality is high, ranging from 40 to 100% [22, 24]. Although the duration of IAP can range from 30 min up to 24 h [25], organ dysfunction can arise as early as 15 min with multi organ failure pending around 5 h. In the random clinical study of Ricardo Lima et al. [26], ACS was induced by packing the abdominal cavity with cotton until the desirable IAP, thus variation in the pressure was measured leading to change in the number of cotton gauze within the abdomen (for example, if pressure was decreased, more gauzes were inserted). In our clinical study, pressure was kept constant allowing us to have stable results.

Autonomic nervous system is influenced, among other factors, by sex hormones. They also exert their effect on vascular permeability to water and protein and the response of vasoactive substances on the vascular system [27]. Therefore, a difference is seen between the genders across the phases of the reproductive cycle. Hence, the theory of blood volume restitution was introduced.

Gonadal steroid hormones, play an important, though opposite, role in the hemodynamic response to hemorrhage. Gender dimorphism in terms of response to acute stress conditions have been reported in several studies, preceding an explanation to the lack of significant difference between the genders as reported in Raju et al. [28].

In our clinical study, there is an apparent superiority in all female groups. The females maintained their MAP at better hemodynamic levels. In addition, they showed their ability to maintain lactate levels.

The reduction in MAP after exposure to IAP of 20 or 30 mmHg was significantly lower in female rats than in male rats. A similar result was reported by Krausz et al. [27] when uncontrolled hemorrhagic shock was induced secondary to massive splenic injury. This was also noted in work completed by Bosch at el [29], where they explained the female gender advantages following trauma-hemorrhage was not solely related to sex but rather to the prevailing hormonal milieu of the victim. In support to the aforementioned statement, no survival benefit is seen in postmenopausal females, as estrogen levels decrease and no myocardial or immune depression was seen in castrated male animals versus non-castrated males [18, 19]. An abundance of evidence highlights the protective effects of estrogens following adverse circulatory conditions as its receptors are expressed to a variety of tissues exerting genomic and non-genomic effects, thus decreasing mortality rates in female. In our study, hormonal status was not evaluated; nevertheless, better hemodynamic results were documented in female rats. Female rats exposed to IAP of 20 or 30 mmHg survived significantly better than male rats exposed to the same pressure.

Clinical and experimental evidence, such as reported in the study by Mark et al. [30] and Kawasaki et al. [31] showed the salutary effects estrogen has in maintaining gut viability following trauma in rats. The beneficial effects of estrogen following trauma induced hemorrhage, as reported by Weniger et al. [32], are seen throughout the human organ systems; ranging from pulmonary and cardiovascular, to the hepatic, gut, and immune system. These effects ultimately impact overall survival. On the other hand, a study by Larsen [33] found that survival advantage for females appears consistent with age suggesting that other components, rather than sexual hormones, play a role in the survival advantage exerted by females.

Blood lactate level of male rats exposed to IAP of 20 mmHg was elevated immediately, whereas in females exposed to the same pressure it was elevated after 2 h only. Although, there was no significant difference regarding the pH between the genders in our current study, the enzymatic processes and glucose production in response to stress and ACS, is initiated by the release of glucocorticoids, glucagon and pituitary hormones and maintained thereafter by the sympathetic nervous system (flight or fight reaction), which is partly related to higher estrogen levels. Mizushima et al. [14] found supporting evidence as female rats showed no depression of cardiac output and hepatocellular function within the first day following severe hemorrhagic shock during the proestrus phase in comparison to males and ovariectomized females. It is thought that estrogens positively influence the restoration of organ function following shock and sepsis, thus contributing to higher survival rates [29]. Nevertheless, the exact mechanism by which the immunomodulatory influence of estrogen remains speculative. Androgens on the other hand cause cardiovascular and immune depression after trauma or other stress related conditions. The consideration of gender and sex hormone status for treatment in the clinical arena represents an important and novel step towards personalized medicine.

Implementation of estrogens in trauma and emergency care require further clinical studies due to the above conflicting results.

In summary, we designed an experimental model for ACS in which pressure was kept constant in rats, based on a saline infusion trough a catheter connected to a balloon within the abdominal cavity. The results indicate that females have a beneficial effect on acute stress conditions, as well as ACS, as they better preserve their physiological and metabolic state. The exact reason for this advantage is still not clear enough.

## Limitations

Our study was not designed according to the proestrus cycle in females nor did it measure the levels of estrogen. This additional information might have spread some light concerning the difference within the female group (before and after menopause) and help emphasize a change within the gender.

## Data Availability

The datasets used and/or analyzed during the current study may be made available from the corresponding author upon request. a_mahajna@rambam.health.gov.il.

## Abbreviations

ACS: abdominal compartment syndrome
IAP: intra-abdominal pressure
MAP: mean arterial pressure
fig: figure
